# Can unreliable auditory hazard warnings help the driver? The effect of timing errors and false alarms on road hazard detection in dynamic road scenes

**DOI:** 10.1186/s41235-026-00718-w

**Published:** 2026-03-17

**Authors:** Jiali Song, Benjamin Wolfe

**Affiliations:** Department of Psychological & Brain Sciences, University of Toronto Mississauga, 3359 Mississauga Rd, Mississauga, ON L5L 1C6 Canada

**Keywords:** Warning cues, Road hazards, Driving, Cue reliability, Attention

## Abstract

Vehicle-based warnings that speed road hazard detection can reduce collision incidence and severity. However, no technology is perfect, and it is critical to understand the impact of incorrect cues on hazard detection. This study, conducted between September 2022 and September 2023, examined the impact of non-spatial hazard warning auditory cues on licensed drivers’ ability to localize hazards (situations requiring immediate response to avoid a collision) in real road footage when cues are mistimed (Experiment 1) and when cues include false alarms (Experiment 2). In Experiment 1, we varied the duration between cue and hazard onset and found that earlier cues speeded responses more than later cues, and warning cues reduced response time regardless of timing. However, each trial included a hazard, whereas hazards are rare on the road. In Experiment 2, we added false alarm warnings and hazard-absent trials in two cue reliability conditions (80% and 50%), and these cues did not significantly affect hazard localization performance regardless of reliability. Although earlier auditory temporal warnings can speed hazard localization, these benefits disappear in the presence of false alarms in attentive drivers and suggest that classic cueing results may not necessarily translate to dynamic natural scenes with ambiguous targets onsets.

**Supplementary Information:**

The online version contains supplementary material available at 10.1186/s41235-026-00718-w.

## Introduction

Safe driving requires responding quickly and accurately to a hazard when one appears. Interventions that speed hazards detection can help drivers respond earlier and reduce the incidence and severity of collisions. How might drivers gain the time they need?

One way to speed response time (RT) in the laboratory is to visually present a spatial cue at the location of an upcoming target (an exogenous cue; Jonides, 1981; Posner, 1980). Typically, detection of a target is faster when the cue correctly predicts its location (valid cue) compared to when the cue appeared in a different location (invalid cue). Previous work indicates that this principle can be applied to driving contexts, as directing a driver’s attention to the location of a hazard using spatial visual cues can speed hazard localization in naturalistic road videos, even when spatial cues were valid only 50% of the time (Wolfe et al., 2021). However, invalid spatial cues drastically decreased the accuracy of hazard localization (Wolfe et al., 2021). Although a real hazard warning system ought to have a higher validity rate than 50%, the catastrophic effects of an erroneous cue may far outweigh the benefits of speeding response time to hazards.

Another way to direct attention in the laboratory is to indicate the timing of target onset. It has been demonstrated in the laboratory that a warning signal reduces RT when it precedes a target requiring a response (Niemi & Näätänen, 1981). The time between the end of the warning signal and the onset of the target (a foreperiod) is thought to allow observers to prepare to respond to an upcoming stimulus (Niemi & Näätänen, 1981). If the same principles apply to the onset of road hazards, warning drivers of an impending hazard before it develops into an immediate hazard should also speed driver response time.

One type of temporal hazard warning commonly found in vehicles today is forward collision warning (FCW) systems, in which the vehicle alerts the driver when a collision is imminent. Studies using police-reported collision data found that FCW reduced crash rates (Cicchino, 2017; Flannagan & Leslie, 2020). An on-road study of driver behavior in FCW-enabled vehicles found that warnings sped response time to low headways; however, drivers also tended to keep a shorter following distances (Zhu et al., 2020). However, FCW systems only respond to imminent collisions, but cannot predict them when they are more subtle (e.g., a hazard hidden behind an obstacle). As such, in some situations or at sufficiently high speeds, warnings may not be provided early enough to help the driver. One simulator study also found that systems that produced warnings after braking has been initiated resulted in poor trust and compliance (Abe & Richardson, 2006), indicating the importance of early warnings.

However, earlier warnings would introduce new problems. As Parasuraman et al. (1997) noted, given the low base rate of collision events, only a small proportion of warnings will represent actual near-collisions, a finding that has been confirmed in natural driving data (Seaman et al., 2022), which would also represent an increase in false alarms. Given that no system can perfectly predict the future, these issues may be exacerbated with earlier warnings and may also be less reliable overall. Therefore, it is important to understand whether drivers can tolerate errors in non-spatial hazard warnings.

Two types of errors are investigated in the current study. The first error type is timing, where the temporal cue may alert the driver to a hazard too early or too late for the driver to respond usefully. Another type is cue reliability, where the temporal cue fails to alert the driver when a hazard is present (miss), and the cue alerts the driver when the hazard is absent (false alarm).

Mistimed warnings are most comparable to auditory warning cues with a variable foreperiod (time between the end of the signal and the target), which have been shown to reduce RT in laboratory studies (Niemi & Näätänen, 1981). However, classic laboratory experiments tend to use discrete, above threshold signals, where it is relatively easy to tell the exact moment the target appears. In contrast, road hazards unfold over time in complex, dynamic environments, and the exact moment of hazard onset may be unclear. These aspects of road hazards have several implications on the effect of warning signals which we will investigate in Experiment 1.

Errors in cue reliability are less comparable to classic foreperiod studies because the foreperiod requires the presence of a warning signal preceding the target event. However, no foreperiods exist during a false alarm, where there is a cue but no target, and during a miss, where there is a target but no cue. Prior laboratory studies investigating target–cue contingency or cue validity on performance found that, when a visual temporal cue has a reliability of 80% (i.e., 80% of cues validly predicts the timing of target onset), a valid cue sped response time by up to 20 ms compared to invalid temporal cues (Coull & Nobre, 1998; Nobre, 2001). Similarly, a prior study using videos of road hazards found that a visual warning cue indicating only the timing of hazard onset (defined as the moment the first visual signal that the situation deviates from normal safe driving) also speeded hazard detection when the cue predicted hazard onset 100% of the time (Wolfe et al., 2021).

However, prior studies have repeatedly demonstrated that false alarms reduce compliance and response rates (the cry wolf effect; Breznitz, 1984). However, other simulator studies suggest that even warning signals with more than a 20% false alarm rate can reduce brake reaction times compared to no-warning baselines (Large et al., 2017; Lees & Lee, 2007; Navarro et al., 2016). These mixed results highlight the need to more closely examine the effect of reliability on cue use.

The current study investigated the extent to which drivers can tolerate auditory warnings cues that err in timing and in validity. Although warning cues can be presented in many modalities, such as visual, auditory, and haptic warnings, the current study focuses on auditory warning cues to isolate the temporal effects of alerting the driver. This approach allows us to avoid added saliency of visual warnings, which may distract the driver.

Participants viewed brief video excerpts of real dashcam footage from the Road Hazard Stimuli dataset (Song et al., 2023), each lasting 1–5 s. After each video, we asked them to localize hazards, defined as objects or events that would have required an immediate response to avoid a collision in an on-road situation. Using temporal annotations from the stimulus set, we inserted auditory cues to warn drivers of upcoming hazards. In Experiment 1, we examined the range of cue-hazard timings that can speed driver response time to hazards. In Experiment 2, we investigated the impact of the reliability of temporal cues on driver hazard detection. Pre-registrations for Experiment 1 (https://osf.io/ekmyh) and Experiment 2 (https://osf.io/94bsc) are available on Open Science Framework (OSF).

## Experiment 1

Experiment 1 investigated the extent to which drivers can tolerate timing errors in auditory warning cues for upcoming hazards. Hazard warning cues with timing errors are akin to a neutral warning signal in a laboratory reaction time study with a variable foreperiod (i.e., the duration of time between the offset of a warning signal and the onset of the target stimulus varies across trials). Typically, in these designs, the shortest foreperiod (i.e., the latest warning) within a block gives rise to the longest RTs compared to other foreperiods (Niemi & Näätänen, 1981).

One reason these laboratory results may not necessarily generalize to the driving context is the gradual onset of the target. Whereas the target in foreperiod studies tend to be discrete (Niemi & Näätänen, 1981), road hazards unfold over time, and the exact moment of hazard onset may be ambiguous. For example, there may be early signals in the scene that allow drivers to predict where a hazard will be (latent hazards; Horswill et al., 2021; Muela et al., 2021; Vlakveld et al., 2011). However, drivers may choose not to respond to these signals, because there are individual differences in how they are interpreted (Horswill et al., 2020; Muela et al., 2021). This temporal aspect of hazards complicates the generalization of laboratory studies.

Moreover, whereas foreperiod studies require a warning signal that precedes the target stimulus, it is possible for an erroneous hazard warning to appear after a hazard is already underway. Because in-car warning systems are inherently reactive (they cannot predict hazard onset), these systems will require some time to process the road scene after the hazard has appeared. To simulate this processing delay in a hypothetical system, we included a condition where the cue occurs 100 ms *after* hazard onset.

Several competing hypotheses can be generated with these considerations in mind. If participants rely on a temporally uncertain cue, warning signals should speed hazard localization, especially for earlier cues. However, if the cue occurs too late, drivers may already have enough information about the scene, which renders the cue unnecessary. In this case, cues should have no effect on RT. Alternatively, a warning signal may even distract the driver from responding to the hazard. In this case, later cues may result in slower RT.

## Methods

### Participant sample

Forty-six participants were recruited from the University of Toronto Mississauga paid participant pool. Drivers were aged 18–25 (mean age = 19.7, SD = 1.71) and had valid driver’s licenses (G2 or full G or equivalent, meaning they have had at least 8 months of road experience), and had a (corrected) near-and-far visual acuity of 20/25 or better in each eye with confirmed using near-and-far ETDRS charts. Based on a power analysis, this sample is able to detect effect with sizes of Cohen’s *dz* of 0.68 with 95% power at *α* = 0.006 after Bonferroni correction for 9 planned pairwise comparisons. Two participants were replaced due to failure to meet the vision requirements. We obtained informed consent from each participant prior to their participation. This study was approved by the University of Toronto Social Sciences, Humanities & Education Research Ethics Board, and adheres to the Tri-Council Policy.

### Stimuli and apparatus

All stimuli were displayed using a computer running MacOS Mojave (10.14.6). The experiment was programmed using PsychToolbox-3 (Brainard, 1997; Kleiner et al., 2007; Pelli, 1997) in Matlab version 2019b. All stimuli were displayed on a 55″ LG OLED TV EF9500 at a viewing distance of 90 cm.

To measure hazard detection performance, segments of footage from Road Hazard Stimuli were shown to participants (Song et al., 2023). Each video contained an annotated hazard that occurred at a variable time at least one second after video start. All hazards required an immediate driver response to avoid a collision and may be vehicles, pedestrians, animals, or other objects on the road. Independent annotators identified the onset time of the hazard in each video, defined as the moment that any visible signal of an impending collision occurs. For example, when encountering a vehicle at an intersection, hazard onset refers to the moment a vehicle starts moving toward the path of travel rather than the moment the vehicle becomes visible, as a stopped vehicle would not cause a collision. Videos ended just before the driver response was visible in the video footage. Only videos with hazards that did not cross the midline of the video were used as stimuli. There were 283 hazard-present videos that met these criteria, with 149 hazards on the left and 134 hazards on the right. For each participant, videos were drawn randomly from this pool without replacement while equating the number of hazards that appeared on the left and right side. Videos were recorded at 720*p* and displayed at a refresh rate of 30 Hz, and extended 50 degrees visual angle (dva) horizontally and 28.2 dva vertically from a viewing distance of 90 cm. To make the localization task less ambiguous, a vertical white line bisected the video into a left and right half whenever the video was visible.

The auditory cue was a series of three pure tones at a frequency of 1800 Hz. Each beep has a duration of 50 ms, and each onset separated by 100 ms. When present, the auditory cue was presented, while the video played at one of four timepoints relative to hazard onset: − 500 ms, − 250 ms, 0 ms, and 100 ms.

### Procedure

**Fig. 1:**
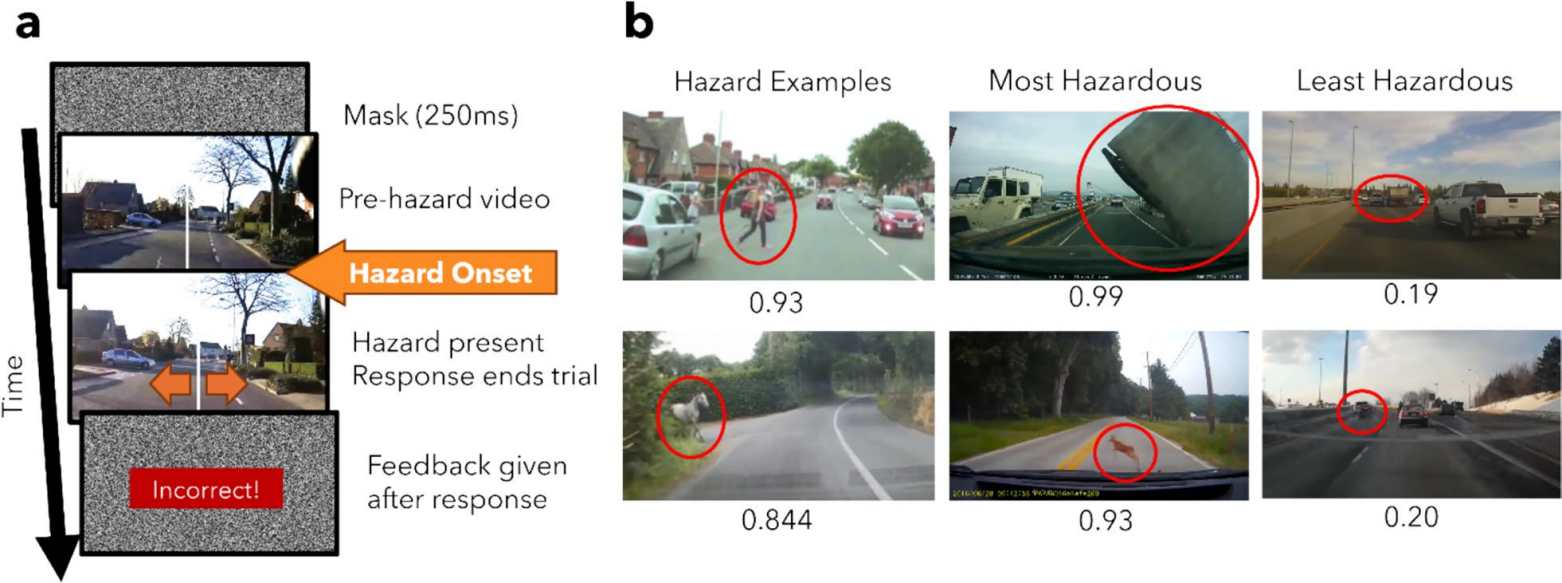
Schematic of stimuli in experiment 1 **a** Schematic of a single trial. Participants fixated on a fixation cross, and then, a mask appeared. Participants watched a segment of video for a random interval of at least 1000 ms before annotated hazard onset, until the participants responded or when the driver in the vehicle responded to the hazard, whichever occurred first. Participants were instructed to localize the target to the left or right of the white line as soon as possible, and free-viewing was permitted during the video. Feedback about timing and accuracy was given after each response. **b** Examples of hazardous videos and associated hazardousness ratings (on a scale from 0 to 1, where 0 is the least hazardous and 1 is the most hazardous) from Road Hazard Stimuli (Song et al., 2023). Red circles highlight the hazard for illustration purposes and did not appear in the actual experiment

We used a cued hazard detection task, modified and adapted from Wolfe et al., (2021). On each trial, as illustrated in Fig. 1a, observers initially looked at a green fixation point for 250 ms, followed by a random dot noise mask that had the same spatial extent as the road video for 250 ms. Then, observers freely viewed a video of a real road scene lasting between one to five seconds. Observers were asked to report whether a hazard, which was present on every trial, appeared on the left or right half of the video using the left and right arrow keys. Observers were instructed to respond as quickly and as accurately as possible, ideally while the video was still playing. The video ended when the observer responded or when the driver in the video responded to the hazard, whichever occurred first. The end of the video was followed by another mask for 250 ms. After they responded, observers received feedback about the accuracy of their responses. Responses were coded as correct if they matched the annotated lateral location of the hazard. The feedback depended on when the participant responded. If they responded faster than 500 ms before the annotated hazard onset, they were shown “Too early!” and if they responded later than two seconds after hazard onset, they were shown “Too slow!” and no feedback was given about their accuracy. If their response was neither too early nor too slow, they were shown “Correct” if they correctly localized the hazard and “Incorrect” if they did not.

The experiment had two blocks, with order counterbalanced across observers. One block was a baseline block with no auditory cues, and the other block contained 5 different cue-hazard stimulus-onset asynchrony (SOA; duration between hazard onset and cue onset) conditions intermixed within the block: − 500 ms, − 250 ms, 0 ms, 100 ms, and no cue onset. Negative times indicate that the cue occurred prior to hazard onset, and positive times indicate that the cue occurred after hazard onset.

To familiarize observers with the task, the experiment began with 10 practice trials with no auditory cues. Prior to each block, observers completed another 10 practice trials. Before the block with auditory cues, the practice trials included all cue onset conditions, whereas the practice trials for the no-cue baseline did not include cues. Each block had 100 experimental trials. In the cued block, each cue onset timing condition had 20 trials, which had an even split of hazards located on the left and right side.

### Data analysis

All data analysis was completed in R 4.1.3 (R Core Team, 2017). Each observer’s data was screened for data quality before being included in the analysis. As per our pre-registration (https://osf.io/ekmyh), observers needed to perform the localization task significantly above chance, as indicated by a binomial test across all responses in all conditions. In addition, observers’ median reaction times, relative to cue onset, must be within 2 s of the annotated hazard onset. All observers met these criteria and were included in the analyses. Because some hazards are more ambiguous than others, any videos where the median response takes place more than 500 ms prior to the annotated hazard onset (i.e., RT below − 500 ms relative to hazard onset) and where participants could not accurately localize the hazard significantly above chance (as indicated by a binomial test) were removed from the analysis for all observers. Based on these criteria, 20 out of 261 videos were excluded (7.6% of all trials), resulting in 241 total videos included in the analysis.

We analyzed RT and accuracy of hazard localization responses. For analyzing RT, only correctly localized hazards were included. Median RT was calculated for each condition and each participant and then averaged across participants. Note that RTs are calculated from annotated hazard onset, and anticipatory responses are accepted up to 500 ms prior, making the RT data approximately normally distributed.

To examine the effect of cue timing on performance, we conducted separate one-way repeated-measures omnibus ANOVAs on accuracy and RT measures. Each ANOVA included cue condition (6 levels of cue onset condition: − 500 ms, − 250 ms, 0 ms, 100 ms, no cue during the cued block, and baseline no-cue block). Additionally, we conducted all pairwise comparisons using Tukey’s HSD test to compare individual cue timing conditions more specifically. Of particular interest are pairwise tests involving the no-cue baseline condition as that will determined whether each cue timing condition significantly increased or decreased response times. Furthermore, each cue condition will be compared with each other to determine whether the differences among cue conditions are meaningful.

## Results

Across all cue timing conditions, reaction times for correctly locating the hazard were significantly faster when an auditory cue was present compared to a no-cue baseline (t >  = 4.5, p <  = 0.001). Correct hazard localization was faster for earlier cues than later cues (e.g., − 500 ms: *M* = 77 ms, SE = 37 ms vs. 0 ms: *M* = 289 ms, SE = 25 ms; t = 6.46, *p* < 0.001, note that mean RTs are from annotated hazard onset and anticipatory responses are accepted up to 500 ms prior).

**Fig. 2:**
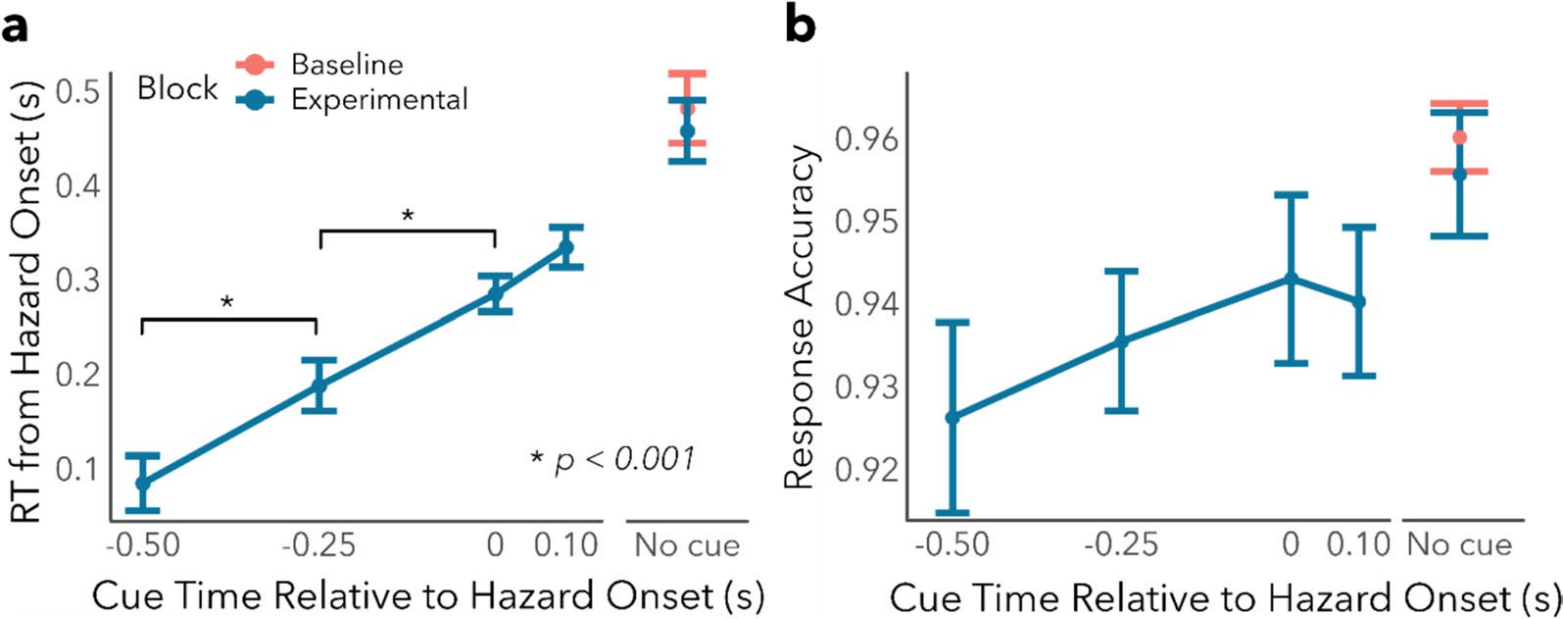
Response time and accuracy as a function of cue onset time. **a** Hazard localization response time from hazard onset plotted as a function of cue onset time from hazard onset. Negative cue times represent early cues that occur before the annotated hazard onset time, and positive cue times represent late cues that occur after the annotated hazard onset time. Error bars represent standard errors of the mean. Orange and turquoise points represent the no-cue baseline and cued experimental conditions, respectively (the no-cue condition was tested once on its own in the baseline block and interleaved with cues in the experimental block). Asterisks represent significant contrasts with *p* < 0.001. Response time increased with later cue onsets, and the presence of a cue regardless of whether it was before or after the annotated hazard time speeded response time compared to no-cue trials. **b** Hazard localization accuracy as a function of cue onset time from hazard onset. Symbol conventions are the same as Fig. 1a. Cue-present trials are associated with a lower localization accuracy compared to no-cue trials, but none of the contrasts were statistically significant

Figure 2a shows RT for correct hazard localization as a function of cue timing conditions. Visual inspection of Fig. 2a suggests that reaction time increased with later cue timings, an observation that was corroborated by quantitative statistical analyses. The one-way within-groups ANOVA comparing RT in each cue timing condition (6 levels) found a significant effect of cue timing (*F*(5,235) = 62.36, *p* < 0.001, *η*^*2*^_G_ = 0.34). To further examine the effect of cue timing, all pairwise comparisons were done using the Tukey method for controlling type I error rate. All pairwise comparisons were statistically significant (*t*(235) >  ± 3.52, *p* < 0.007, see Supplementary Table 1 for full statistics), except for the contrast between 0 and 100 ms cue onset conditions (*t*(235) =  − 1.791, *p* = 0.47) and the contrast between no-cue in the baseline block and no-cue in the experimental block (*t*(235) =  − 0.864, *p* = 0.95). Overall, these results suggest that a timely alert can reduce response time compared to no alerts, and earlier alerts reduce RT more than later alerts.

Figure 2b shows hazard localization accuracy as a function of cue timing conditions. Although accuracy remained above 90% across all conditions, visual inspection of Fig. 2b indicates that earlier cues reduce accuracy compared to no-cue trials. The one-way within-groups ANOVA comparing accuracy in each cue timing condition found a significant main effect of cue timing (*F*(5,235) = 2.722, *p* = 0.02, *η*^*2*^_G_ = 0.036). However, all pairwise analyses showed that this main effect of cue timing was mainly driven by the difference between the − 0.5 s cue onset time and the no-cue condition in the baseline block (*t*(235) =  − 3.132, *p* = 0.02), and no other comparisons were significant (*t*(235) <  ± 2.72, *p* > 0.08, see Supplementary Table 2 for more details). This size of this reduction in accuracy was small, at approximately 3%. Overall, these results indicate that performance accuracy remained high across cue timing conditions.

## Discussion

Experiment 1 investigated whether an auditory temporal cue could speed hazard detection. Across all cue timing conditions, mean hazard localization accuracy exceeded 90%. Importantly, reaction times were significantly faster when a cue was present compared to a no-cue baseline. Hazards were localized faster when cues preceded the hazard, with the fastest reaction times in the − 500 ms condition (− 500 ms: *M* = 77 ms, SE = 37 ms vs. 0 ms: *M* = 289 ms, SE = 25 ms; *t* = 6.46, *p* < 0.001), suggesting that participants are tolerant to timing errors in warning cues. Additionally, although response time was slower in the 100 ms cue timing condition compared to other conditions, these results suggest that earlier non-spatial auditory cues (up to 500 ms before the hazardous situation) help to orient attention to road hazards, even in the absence of specific location information in the cue. Furthermore, the earlier the cue, the earlier the response, with minimal decrement in localization accuracy.

The results of Experiment 1 are broadly consistent with classic variable foreperiod findings, where the shortest foreperiod (the latest warning onset) produced the slowest RT, and RT decreases as the foreperiod duration increases, particularly with a short mean foreperiod (mean of 162.5 ms in the current experiment) and a relatively small range (Niemi & Näätänen, 1981). This is consistent with our result that hazard localization speed roughly increased linearly with later warning onset time. Furthermore, all cued conditions had faster RTs than all no-cued conditions, even when the cue started after hazard onset. This result is consistent with the idea that warning cues have an overall alerting effect, particularly when they occur near the time of target onset (Niemi & Näätänen, 1981).

We also reported very short RTs relative to hazard onset, particularly at the earliest cue onset condition (500 ms before hazard onset), which would be impossible for classic foreperiod studies. This is due to the gradual unfolding of road hazards, and participants are likely able to predict the location of the hazard based on the presence of latent hazards and the structure of the road scene (Horswill et al., 2020; Muela et al., 2021). These results indicate that, in the presence of highly accurate, early warning cues, participants were able to respond to early signs of hazards, which may subsequently reduce collision incidence and severity on the road.

One important caveat for interpreting these data is that all trials contained a hazard, and therefore, all auditory cues were 100% valid. Since there was a hazard on every trial, participants had an expectation that they would need to respond any time an auditory cue occurred. This expectation that a hazard is present may have biased them to respond at earlier times even though the evidence they have collected about the hazard is uncertain. The small decrement (approximately 3%) in localization accuracy may reflect such uncertainty, but such a decrement not necessarily be meaningful in a real road context. If drivers prioritize braking as the primary response when facing uncertainty about hazard location, earlier braking would still reduce the severity of the collision or avoid a collision entire.

Additionally, on the road, in-vehicle hazard warning systems are unlikely to be 100% accurate. In the current study, cue onset rates reflect a hazard warning system that correctly detects the hazard 80% of the time and miss the hazard 20% of the time. However, real computerized systems are not likely to only miss the hazards, and they will also produce false alarms, where a warning cue is presented in the absence of a hazard. False alarms present a separate, more concerning type of error that drivers must contend with on the road. We examine the effect of reliability and false alarms in Experiment 2.

## Experiment 2

In Experiment 1, a hazard was present on every single trial, so cue onset could perfectly predict hazard onset, even though the cue also was absent on 20% of the trials (misses). In the current experiment, we investigated what happens when cues indicate a hazard in the absence of a hazard (false alarms). To enable false alarm warning cues, we added hazard-absent trials to the experiment such that only half of all trials contained a hazard, resulting in a hazard prevalence of 50%. Such a design also allows us to vary the reliability of the cue in both hazard-present and hazard-absent situations.

Previous work that examined the effect of visual–spatial cueing on road hazard detection found effects that are consistent with laboratory spatial cueing effects (B. Wolfe et al., 2021). Even when a spatial cue with a reliability of only 50% (the cue was valid, or correctly indicated hazard location, 50% of the time), hazard detection RT was significantly reduced on trials with a valid cue compared to a no-cue baseline. These results suggest that classic spatial cueing effects found in the laboratory can be applicable in a road hazard detection context. The same study also reported that an 100% reliable temporal visual cue can also speed response time compared to a no-cue baseline (B. Wolfe et al., 2019). However, it is unclear whether a non-spatial temporal cue can maintain its effectiveness at reducing hazard detection RT at lower reliability similar to a spatial cue.

Prior laboratory studies using simpler stimuli have shown that a temporal visual cue that is valid 80% of the time could speed reaction time when the target appeared shortly after the cue (Coull & Nobre, 1998; Olk, 2014). Moreover, in laboratory paradigms using visual cues, the reliability of a cue (the proportion of cues that are valid) positively correlated with the size of the cue validity effect (Arjona et al., 2016; Lou et al., 2022). Based on these results, one would also expect the effect of cue validity to be positively related to reliability in a road hazard localization context.

In Experiment 2, we investigated the hypothesis that the effect of trial validity would increase when cue reliability increases in a similar road hazard localization task as in Experiment 1. We selected 0 ms cue onset condition in this experiment because Experiment 1 showed that it speeded localization accuracy with the least decrement to detection accuracy. Specifically, we expected that the presence of a warning cue when a hazard is present (valid trial) should speed response time, and the absent of a warning cue when a hazard is present (invalid trial) should slow response time compared to a no-cue baseline, regardless of reliability. However, when a cue is present when the hazard is absent (an invalid trial with a false alarm), RT and error rate of hazard localization should increase.

We manipulated reliability by varying the proportion of valid cues across two groups. One group received low reliability cues (50% valid trials), whereas the other group received high reliability cues (80% valid trials), similar to reliability conditions commonly used in laboratory cueing paradigms (Arjona et al., 2016; Lou et al., 2022). We expected valid trials to speed hazard localization RT and invalid trials to slow localization RT, compared to a no-cue baseline. If participants are sensitive to overall cue reliability, we expected the effect of trial validity to be larger for a high reliability cue compared to a low reliability cue.

The presence of auditory cues may also have a general alerting effect compared to trials on which the auditory cue was not present. If this is true, then observers should response faster on trials with auditory cues compared to trials without auditory cues regardless of whether the trials were valid. We were also interested in an order effect because participants may be more fatigued during the second block which may interact with the effectiveness of warning cues. For brevity, we summarize these results in the discussion section. The detailed methods and results of these analyses are available in the Supplementary Materials.

## Methods

### Sample

We recruited a new sample of 48 licensed drivers (G2, G, or equivalent) aged 18–36 (mean age = 21.12, SD = 3.2) with (corrected) near-and-far visual acuity of 20/25 or better in each eye confirmed using near-and-far ETDRS charts. We determined our sample size based on a power analysis which indicated that we can detect an effect size (Cohen’s *f*) of 0.288 at a Type-I error rate of 0.05 and power of 0.95, which is the smallest effect size of interest based on pilot data (see our pre-registration https://osf.io/94bsc). Two participants were replaced due to software errors. All recruitment procedures were the same as Experiment 1.

### Procedure

The same set of videos from Experiment 1 was used. In addition, videos that did not contain a near-collision (i.e., a hazard) were also included in the experiment. Videos were randomly drawn from a pool of 270 unique hazard videos and 419 unique non-hazard videos. All videos came from Road Hazard Stimuli (Song et al., 2023) and were shown using the same setup as Experiment 1.

**Fig. 3:**
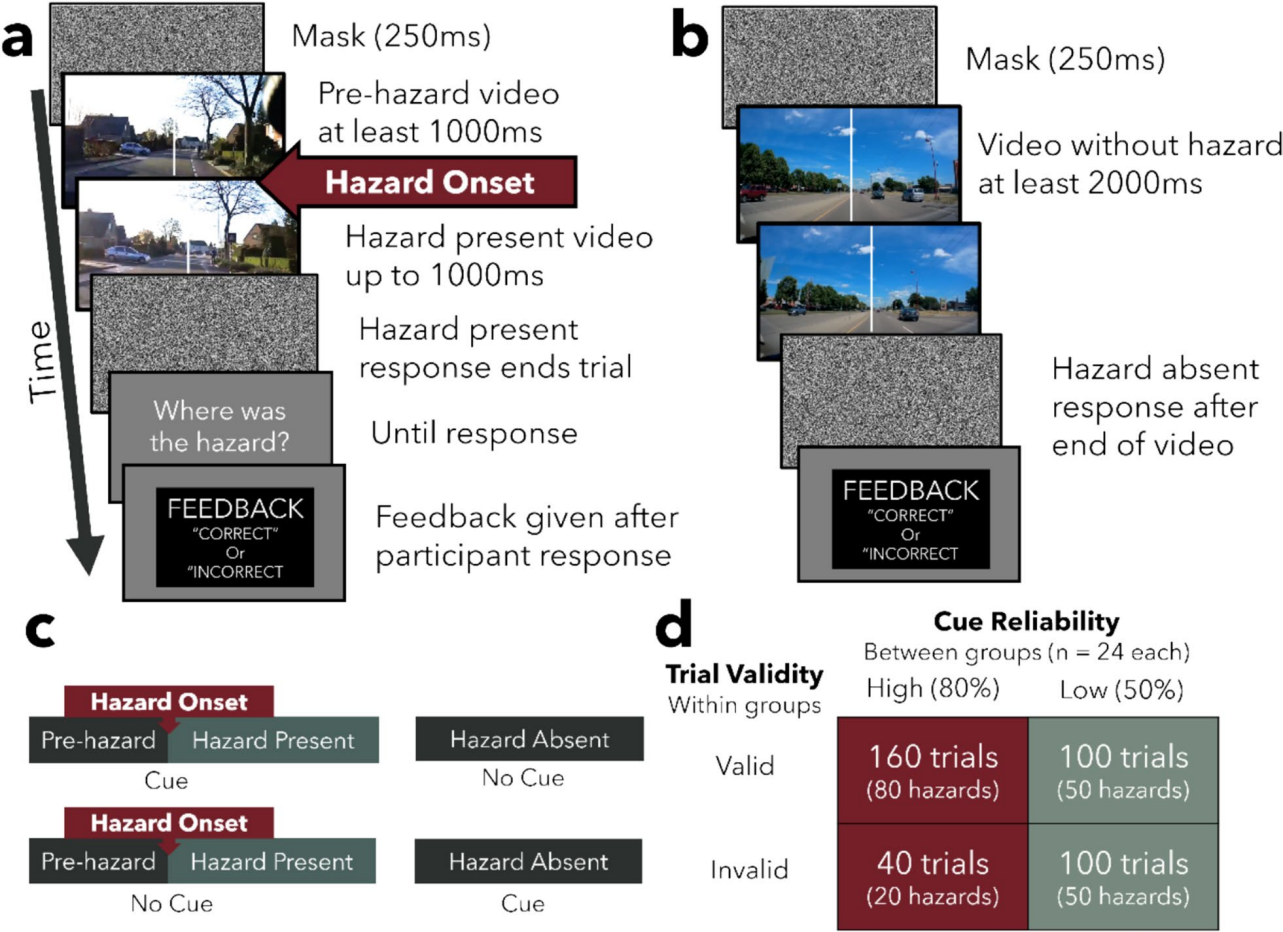
Schematic of experiment 2 design*.* **a** Schematic of a hazard-present trial. **b** Schematic of a hazard-absent trial. **c** The top and bottom rows are schematics of valid and invalid cue trials, respectively. The left figures represent hazard-present trials, and the right represents hazard-absent trials. **d** Table of trial counts in each condition. For the high cue reliability group, 80% of trials were valid, whereas for the low reliability group, only 50% of the trials were valid. Cues were present on 50% of all trials, and 50% of all trials included a hazard

Figure 3 shows a schematic view of each trial in each condition. On each trial, participants watched brief road videos with durations between one and five seconds and reported hazards which required an immediate response in an on-road situation (hazard detection) by pressing the “5” key on a keyboard numpad as quickly as possible. Video presentation stopped when the participant responded or until the end of the video, whichever was earlier. If participants indicated that a hazard was present, they were then asked to indicate whether the hazard occurred on the left or right side of the screen using the “4” and “6” keys on the numpad (hazard localization). On 50% of the trials, the video did not contain such a road hazard, and participants indicated the absence of a hazard by pressing the “8” key on the keyboard numpad after the end of the video. Detection and localization accuracy feedback was provided after each trial. Localization responses were untimed, and only the detection responses were used for analyses of RT. At the beginning of the experiment, all participants completed 20 practice trials. The first 10 trials asked participants to only detect the hazard, and the second 10 practice trials asked participants to localize the hazard if they detected a hazard.

For hazard-present trials where an auditory cue was presented, cue onset was always concurrent with the annotated hazard onset. This the 0 ms cue onset condition was chosen because Experiment 1 found speeded RT while localization accuracy was also the highest of all cue onset conditions. For hazard-absent trials where a cue was present (invalid trials with false alarms), the cue was presented at a random time within the video that followed the same bounding parameters as hazard onsets (at least 1 s after video onset and 1 s before the end of the video).

Hazard presence (hazard present or absent) and trial validity (valid, invalid, and no-cue baseline) were manipulated within groups. Trial validity depended on hazard and cue presence. On hazard-present trials, valid trials included an auditory cue, and on invalid trials, no cues were presented. On hazard-absent trials, valid trials did not have an auditory cue, whereas invalid trials had an auditory cue. Valid and invalid trials were randomly intermixed within the cued block. As a baseline measure, a separate block was also conducted where no cues appear on any trials. Each block began with 20 practice trials, followed by 200 experimental trials. Block order was counterbalanced across participants.

To examine the effect of cue reliability, we varied the percentage of valid and invalid trials between two groups to which participants were randomly assigned. Although an auditory cue was always present on 50% of the trials in the cued block (100 trials), the high reliability group received valid trials 80% of the time (i.e., cues accurately reflected video content 4/5 times, on 80 trials), whereas the low reliability group received valid trials 50% of the time (i.e., cues independent from video content on 50 trials). Valid trials were divided equally between hazard-absent and hazard-present trials, and the location of the hazard was balanced within each block.

### Data analysis

As per our pre-registration (https://osf.io/94bsc), the inclusion criteria were identical to Experiment 1. Additionally, we excluded any videos from the analysis for which detection performance was not significantly higher than chance level, as determined by a one-tailed binomial test. We also removed hazard-present videos where the median detection RT was faster than 0.5 s before hazard onset time. This procedure removed 18 hazard videos (6.9% of hazard videos and 5.8% of all trials), resulting in 510 total videos included in the analysis. For RT analyses, only trials in which the hazard was correctly detected and localized were included.

Hazard-present and hazard-absent trials were analyzed separately. RTs for hazard-present videos started at hazard onset, whereas RTs for hazard-absent videos started at the end of the video, since no hazard was present in these videos. For hazard-present trials, we analyzed hazard detection RT, hazard detection accuracy, and hazard localization accuracy. For hazard-absent trials, participants were not asked to localize a hazard if they did not detect one, and so only hazard detection RT and accuracy were analyzed.

To examine the effect of trial validity, we conducted separate 2 × 3 mixed factorial ANOVAs on detection RT and accuracy with cue reliability (high and low) as a between groups factor, and trial validity (valid, invalid, no cue) as a within-groups factor. The main effect of trial validity from each ANOVA tested whether there was an overall cueing effect, indicating whether participants benefitted from cues at all. The interaction between trial validity and cue reliability indicated whether participants were sensitive to cue reliability effects. All pairwise comparisons were evaluated using the Tukey HSD to evaluate the effects of valid and invalid trials compared to the no-cue baseline.

## Results

**Fig. 4:**
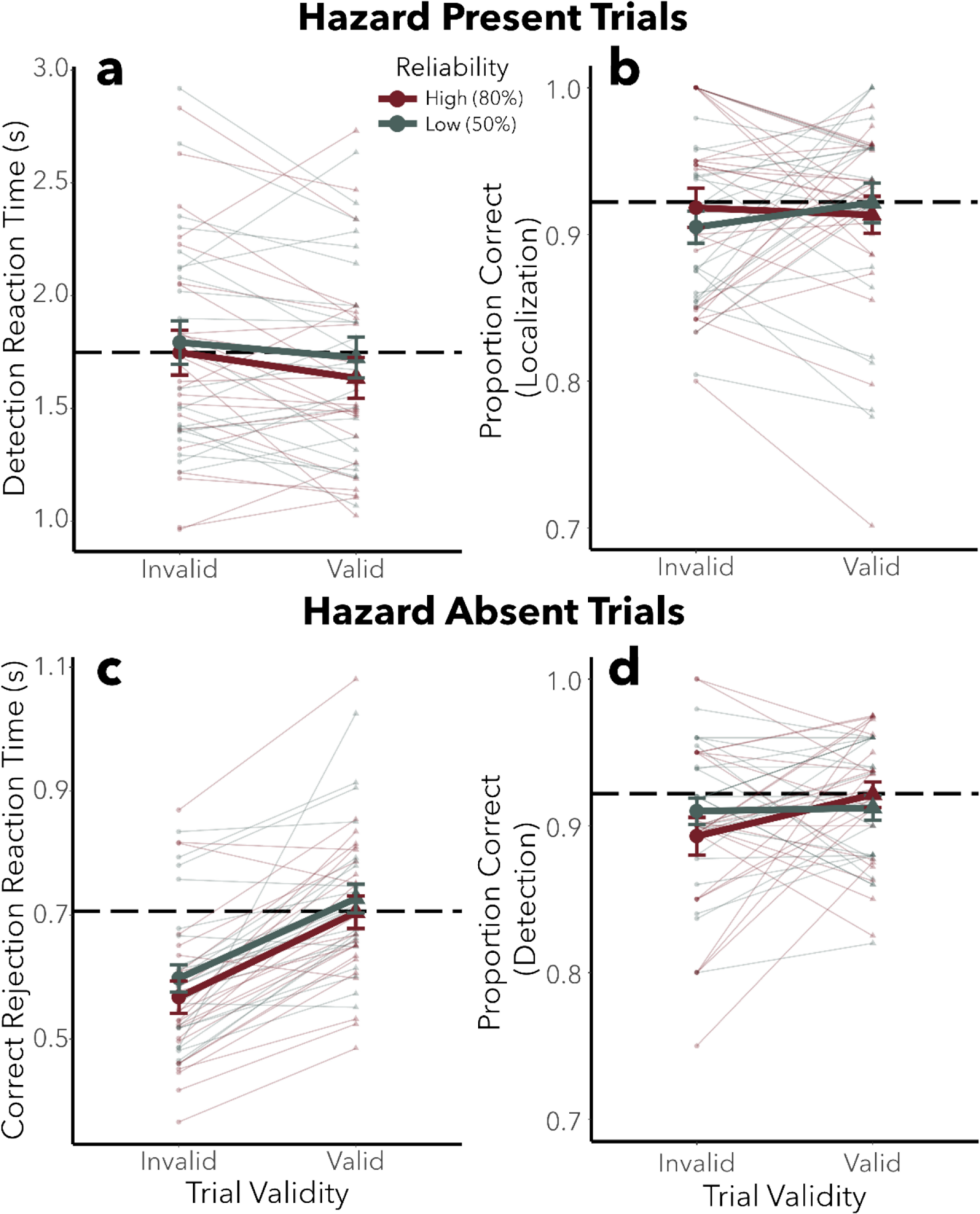
Hazard detection response time and accuracy as a function of trial validity and reliability. **a** Detection response time of correctly localized, hazard-present trials as a function of reliability and trial validity, relative to hazard onset. **b** Proportion of correctly localized, hazard-present trials as a function of reliability and trial validity. **c** Detection response time of correctly detected hazard-absent trials as a function of reliability and trial validity, relative to the end of the video. The presence of a cue (invalid condition) on hazard-absent trials significantly decreased reaction time compared to hazard-absent trials without a cue (valid condition). **d** Proportion of correctly detected hazard-absent trials as a function of reliability and trial validity. There were no other significant effects of cue reliability of validity for any measures shown in Fig. 4. Error bars correspond to ± 1 standard error of the mean, and lines represent individual participants. The black dotted line represents the performance in the no-cue baseline block

Figure 4a and 4b shows the effect of trial validity and reliability on RT and accuracy, respectively, for hazard-present trials. Visual inspection of Fig. 4a and 4b suggests that performance stayed near the level of the no-cue baseline block (horizontal dotted line) in all conditions. The omnibus ANOVAs corroborated these observations and found no significant effects of cue reliability or validity on RT or accuracy on hazard-present trials (*F* ≤ 2.477, *p* ≥ 0.09, *η*^*2*^_*G*_ ≤ 0.009; see Supplementary Table 3 for full statistics). Given the null effect of cue validity, a Bayesian analysis was conducted to examine the strength of the evidence against the null hypothesis, which yielded a Bayes factor of 0.053 for response accuracy and 0.081 for response time (see Supplementary Materials for details on this analysis). This analysis indicates that the data support the null hypothesis that there is no validity effect.

Figure 4c and 4d shows the effect of trial validity and reliability on RT and accuracy, respectively, for hazard-absent trials. Visual inspection of Fig. 4c indicates that RT was faster on invalid trials when there was a cue present but no hazard, than on valid trials when there was no cue present and no hazard. The analysis of variance found a significant effect of trial validity on RT (*F*(2,92) = 46.331, *p* < 0.001,* η*^*2*^_*G*_ = 0.226). A subsequent simple main-effects analysis found a significant difference between valid and invalid conditions (*F*(1,47) = 82.339, *p* < 0.001, *η*^*2*^_*G*_ = 0.247) and a significant difference between invalid and no-cue baseline conditions (*F*(1,47) = 50.236, *p* < 0.001, *η*^*2*^_*G*_ = 0.232). The valid condition did not significantly differ from the no-cue baseline condition (*F*(1,47) = 0.779, *p* = 0.38, *η*^*2*^_*G*_ = 0.003).

Inspection of Fig. 4b indicates that proportion correct was slightly lower than baseline for cued trials. The ANOVA on accuracy found a significant main effect of trial validity (*F*(2,92) = 3.761, *p* = 0.03, *η*^*2*^_*G*_ = 0.043). Further simple main-effects analyses corroborated this result and found that valid trials differed significantly from no-cue baseline (*F*(1,47) = 5.964, *p* = 0.02, *η*^*2*^_*G*_ = 0.055), though the effect size is very small as the mean difference between these conditions is 1.6%. No other simple main effects were significant (*F*(1,47) ≤ 3.085, *p* ≥ 0.09, *η*^*2*^_*G*_ ≤ 0.024).

In summary, we found an effect of trial validity for hazard-absent trials. The presence of an auditory cue (although invalid), speeded reaction time but slightly decreased accuracy compared to baseline. There was no evidence of a benefit of cueing on hazard-present trials.

## Discussion

In Experiment 2, we investigated the extent to which participants can tolerate poor reliability in auditory hazard warnings. We found that regardless of the reliability, cues did not significantly impact participants’ ability to detect hazards. Furthermore, the effect of cueing remained minimal regardless of their perceived hazardousness (see Supplementary Materials for details), indicating that even in ambiguous situations, drivers did not use unreliable cues. However, we found faster RT and higher detection accuracy for high-rated hazards compared to low-rated hazards, suggesting that high-rated hazards may be less ambiguous and easier to detect than low-rated hazards.

Our finding that hazard detection speed (on hazard present trials) was not affected by cues regardless of reliability are contrary to previous laboratory studies that reported that 80% valid temporal cues produced a cue validity effect (Coull & Nobre, 1998; Nobre, 2001; Olk, 2014). There are several differences between the current study and the prior work discussed above. In these laboratory studies, cues preceded the target by at least 300 ms, whereas cue onset was concurrent with hazard onset. The results of Experiment 1 indicate that even a 0 ms cue onset could speed response time, which suggests that a concurrent cue is not too late to benefit hazard detection. Another difference is that a cue was only present on half of the trials in the current experiment, whereas in prior laboratory experiments, a cue was present on every trial regardless of reliability (Coull & Nobre, 1998; Nobre, 2001; Olk, 2014), whereas for the current study, the cue was not present on every trial.

The absence of a cue in safe situations reflects a constraint of in-vehicle implementation. Because road hazards unfold over time and monitoring the road situation is done continuously, it is unclear when a “safe” signal should be given to the drivers. Furthermore, an implementation of a “safe” signal will likely annoy the driver which may cause them to disable a hazard cueing system altogether (Parasuraman et al., 1997). This makes a design in which a cue is always present on every trial highly artificial and unrealistic for on-road situations. This difference in design may have exaggerated the alerting effect of the auditory cues used in the current experiment, an idea that is consistent with the significant decrease in reaction time when a cue occurred during hazard-absent trials. Our finding that participants ignored valid cues even when hazards were present suggests that a simple alerting effect does not necessarily speed hazard detection. However, participants may have habituated to the cues and ignored them over time.

Given that hazard localization performance was high (the average accuracy was 93%), a ceiling effect may have prevented participants from performing better at the task when a valid cue was present. In a more difficult task where hazards were not obvious or detection was near threshold, or when participants are distracted, cues may be more beneficial to hazard detection performance. Despite the inherent timing ambiguity of dynamic road hazards, drivers may be good at ignoring extraneous sources of non-spatial temporal information.

## General discussion

Overall, our results indicate that in a laboratory setting, non-spatial auditory warning cues that have a high false alarm frequency are unlikely to be helpful. Although Experiment 1 found that an early warning signal can speed hazard detection time by approximately 400 ms, even when it is only 80% reliable, the result is tempered by the fact that, in Experiment 2, when warning cue errors included false alarms, participants did not benefit from the cues regardless of cue reliability. These results suggest that hazard localization performance is more robust against mistimed warning signals than errors in cue occurrence (particularly false alarms).

In terms of reliability, both Experiments 1 and 2 included a comparable high reliability condition where warning cues were 80% valid. Whereas valid cues speeded correct localization RT in Experiment 1, valid cues failed to meaningfully affect hazard localization performance in Experiment 2. There are two major differences between Experiment 1 and Experiment 2 that would have likely contributed to this difference, the presence of false alarms, and hazard prevalence, which we discuss further below.

### False alarms and use of warning cues

A key type of errors introduced in Experiment 2 was false alarms. Experiment 1 included only misses (hazard-present and cue-absent trials), but invalid cues in Experiment 2 included both misses and false alarms (hazard-absent and cue-present trials). Operators are likely to ignore a system that generates too many false alarms and may even negatively impact response to critical events (Breznitz, 1984; Dixon et al., 2007). In the context of in-vehicle collision warnings more specifically, simulator studies have found that systems that are more prone to generate false alarms decrease driver compliance (Bliss & Acton, 2003; Wickens & Dixon, 2007) and may even increase latency of responses to critical events (Cummings et al., 2007). Our results align well with the idea that inflated false alarm rates decrease compliance.

However, our finding in Experiment 2 that participants ignored cues in both high and low reliability conditions runs in contrary to the results of prior studies that found a correlation between performance and reliability (Bliss & Acton, 2003; Wickens & Dixon, 2007). Moreover, other simulator studies on imperfect collision warnings found similar compliance rates despite varying false alarms rates (Maltz & Shinar, 2004; Schwarz & Fastenmeier, 2017), and one study even reported benefits of false alarms relative to perfect alarms as ignoring an unreliable system meant participants were less affected by a system miss in a critical situation (Fu et al., 2019).

One possible explanation for the discrepancy between the current results and the literature is the number of false alarms. Whereas prior studies (Fu et al., 2019; Maltz & Shinar, 2004; Schwarz & Fastenmeier, 2017) had only 8 or fewer incidences of false alarms in one experimental session, our study included at minimum 20 incidences of false alarms in one experimental session. Reliability rates in the current study are comparable in range or even higher than those tested in simulator studies, owing to the increased number of total trials/events participants needed to respond to. It may be that there are a critical number of false alarms that participants are willing to tolerate in the duration of a single study session, and that 20 is well beyond the critical number. Other studies suggest that the credibility of a system can be restored if participants believe there is a plausible reason for the system to generate a false alarm. In these cases, alarms are not false alarms, but unnecessary alarms (Lees & Lee, 2007). Unnecessary alarms have a smaller impact on drivers’ compliance with the warning system than false alarms, and systems that make unnecessary alarms tend to be evaluated more positively than systems that make false alarms (Kaß et al., 2018; Lees & Lee, 2007; Naujoks et al., 2016).

However, the difference between false and unnecessary alarms is one of the interpretations. A driver can interpret the same warning cue as unnecessary if they can believe that there is a plausible reason for the system to generate a warning, or a false alarm if they cannot. This may explain the difference between the current results vs prior simulator studies. In simulator studies, road scenarios are continuous, and it is less clear when a situation ends and another begins. Such uncertainty may have led participants in simulator studies to interpret false alarms as unnecessary alarms even though the hazard does not materialize. Given the relatively small number of critical situations tested in simulator studies, interpreting a proportion of false alarms as unnecessary alarms may have resulted in a measurable reduction in hazard response time and increase in warning compliance compared to the baseline condition without warnings.

### Hazard prevalence and use of warning cues

Another important difference between Experiments 1 and 2 is hazard prevalence. Experiment 1 used 100% hazard prevalence, and Experiment 2 used 50% hazard prevalence. The most comparable conditions are the 0 ms SOA condition in Experiment 1, and the valid cue condition on a hazard-present trial in Experiment 2, since both types of trials include a warning cue at the time of hazard onset, and a hazard was present on the trial. Numerically, the response times observed in Experiment 2 (mean RT = 1.67 s) are much longer than the comparable condition in Experiment 1 (mean RT = 0.28 s). This result is consistent with findings the foreperiod literature that when the overall occurrence of the target decreases, RT also increases (Niemi & Näätänen, 1981). This suggests that participants are sensitive to the contingency between a cue and a target even when the target is unfolds over time in a dynamic scene.

However, the low prevalence effect is known to inflate miss rates in the laboratory and in more naturalistic situations such as medical imaging and baggage screening (Wolfe et al., 2007). Similar low prevalence effects have been found in the context of road hazard detected in simulator (Beanland et al., 2014) and in a computer task in the laboratory (Kosovicheva et al., 2023). These prevalence effects tend to be larger at more extreme lower prevalences than those tested here. The fact that localization accuracy in comparable conditions remained high in Experiment 1 (94%) and 2 (92%) suggests that 100% and 50% prevalences were high enough to avoid the low prevalence effect. However, it is not clear how prevalence interacts with cue compliance.

First, the higher number of total situations encountered in a computerized study compared to simulator studies means that participants also experienced a higher number of unreliable cues, which may signal more strongly that the warning system is unreliable. Rather than a critical false alarm rate, there may be a critical number of false alarms per session that drivers accept before ignoring the warning system (Breznitz, 1984). The results of the current study suggest that the critical number is fewer than 20 trials, but it is possible that the critical number depends on total hazard prevalence and should be investigated in future work.

Taken together, our findings suggest that participants are sensitive to the prevalence of hazards and the presence of false alarms when deciding whether to rely on warning cues in a hazard detection context. These findings highlight several factors of experiment design that affects the information drivers rely on to detect hazards in dynamic scenes and suggest that drivers may be good at ignoring extraneous auditory information in this task.

### Limitations and future directions

We chose the Road Hazard Stimuli because it is a naturalistic dataset, as all videos came from real road scenarios crowd-sourced from social media (Song et al., 2023). However, several aspects of the design of the study affect its generalizability. For one, all hazard prevalence rates tested in the current study were extremely high compared to real road scenarios. Laboratory studies require enough trials to accurately estimate performance, so the probability of hazardous situations tends to be inflated compared to the road. Drivers are extremely unlikely to encounter 100 collisions or near-collisions within a single hour. The high hazard prevalence rates used in this study created an expectation of needing to make responses to road hazards. Given the low probability of hazards occurring in real driving, RTs in the context of hazard warnings on the road are likely to be longer than what we observe in laboratory. This makes the results of the current study an optimistic estimate of how drivers may behave on the road.

Another limit to generalizability is that the laboratory environment is much more controlled than vehicle cabins in real on-road situations. Participants in the current study are likely to be much more alert and attentive to the task at hand (searching for hazards) than a driver on the road. Aside from operating the vehicle, drivers often need to engage in multitasking, such as deciphering a map to navigate, or having a conversation with a passenger. In these less optimal conditions, non-spatial auditory cues may produce benefits (Arslanyilmaz, 2020; Lees & Lee, 2007). Furthermore, each trial is only a few seconds long, which is a much shorter duration in which a driver must be attentive than typical driving. At prolonged durations, drivers are likely to experience vigilance decrement, which is likely exaggerated during automated driving (Biondi et al., 2024; Greenlee et al., 2024; Lin et al., 2025; McWilliams & Ward, 2021). It is possible that auditory non-spatial warnings would be more effective when vigilance is poor; however, the interaction with system trust should be considered carefully. Future research should investigate how cue reliability affects cue use under less optimal conditions with reduced baseline performance.

The current experiments only tested proximate hazards—situations that require immediate response to avoid collision or near-collision. On the road, latent hazards are likely more common (Vlakveld et al., 2011). Drivers learn to detect and predict these subtle hazards with road experience; expert drivers are much better than novice drivers at identifying and preemptively responding to latent hazards to avoid potential collisions (Borowsky et al., 2010; Horswill et al., 2021; Sagberg & Bjørnskau, 2006). Early warnings can, conceivably, alert drivers to latent hazards to promote early detection. However, most latent hazards do not develop into immediate hazards, elevating the likelihood of false alarms in the context of latent hazard warnings. Frequent false alarms decreases warning compliance and increase annoyance, which may result in turning off the system altogether (Bliss & Acton, 2003; Lees & Lee, 2007; Parasuraman et al., 1997). Moreover, when multiple latent hazards are present in the scene, driver attention could be drawn toward the wrong latent hazard, which would distract the driver from an actual immediate hazard developing in the scene. For these reasons, the trade-off between warning timing and accuracy is particularly tricky for latent hazards.

Another relevant consideration that tempers our findings is the trust in warning technology compared to a laboratory environment. In the laboratory, there may be an overall a lack of trust in the experimental situation. Participants in the current study knew they were participating in a psychology experiment, and expectations about psychology experiments may have resulted in distrust of the cues leading participants to ignore the cues. On the road, this may not be the case, as drivers would be able to choose whether to use such an auditory warning system, and those who choose to use it likely do because they believe that the warnings are reliable. For these reasons, drivers interacting with such systems on the road may be more likely to comply with the warnings than in an experimental psychology laboratory setting.

In applied situations, some skepticism may be beneficial because overtrust in technology is particularly detrimental when the technology’s limitations are poorly understood (Fu et al., 2019; Lee & See, 2004; Manchon et al., 2022; Payre et al., 2016). For example, failure to monitor the environment while an autonomous vehicle is engaged in autonomous driving could result in a failure to take over when required (during disengagement), and a collision may occur (Fu et al., 2019). In road safety contexts, it is particularly important for drivers to understand the capability and limitations of their car’s technology so they can be prepared to respond in the event of a critical failure (Dixit et al., 2016; Hancock et al., 2020; Khattak et al., 2021).

Furthermore, several differences between the warnings used in the current study and real warning design limit the generalizability of these findings. First, real categorization systems implemented in vehicles may output graded warnings that indicate the urgency of the situations. For instance, one medium-fidelity simulator studies have found that graded alerts lower the rate of inappropriate responses to unnecessary warnings and were more trusted than all-or-nothing, single-stage warnings (Lee et al., 2004). Another low-fidelity simulation of three-level forward time-to-collision warning improved sustained visual attention compared to a two-level system, but it is unclear how this compares to a single-stage system (Shariatmadari et al., 2025). However, both studies were conducted in the context of distracted driving and may not apply to the attentive conditions of the current study. Furthermore, hazards tested were lead-vehicle braking events, and it is unclear the extent to which these results apply to the more ambiguous situations tested in the current study. Lastly, these in-vehicle warning systems are often multimodal, involving visual auditory and haptic warnings rather than auditory only. Multimodal alerts may capture attention more strongly than the auditory-only intervention used here and may be more effective at directing attention. More research is required to elucidate how these factors of warning design affect drivers’ ability to respond to hazards.

In sum, we found that drivers were tolerant to variations in the timing of warning cue onset and omission errors, but they ignore warning cues when warning cues included false alarms. These results suggest that effects of cue reliability commonly used in laboratory studies with simple stimuli may not necessarily translate to the constraints of more complex ecological situations such as road hazard detection and must be tested in the relevant contexts with the appropriate task demands before being applied to real-world situations.

## Data availablity

Experiment 1’s pre-registration is available on Open Science Framework (OSF) at https://osf.io/ekmyh. Data and materials are available on OSF at https://osf.io/yhu3j. Experiment 2’s pre-registration can be found on OSF https://osf.io/94bsc. Data and materials for Experiment 2 are available on OSF at https://osf.io/bdfqc. The stimuli are available at https://osf.io/tgzb7.

## Supplementary Information

Additional file1 (PDF 377 KB)

## Abbreviations

RT: Response time
SOA: Stimulus-onset asynchrony
