# Supplementary material for: Can unreliable auditory hazard warnings help the driver? The effect of timing errors and false alarms on road hazard detection in dynamic road scenes
Source: Cogn Res Princ Implic. 2026 Mar 17;11:24. doi: 10.1186/s41235-026-00718-w (PMC12996480; doi:10.1186/s41235-026-00718-w)
Supplement: Supplementary file 1 — Additional file1 (PDF 377 KB) [file 41235_2026_718_MOESM1_ESM.pdf]

Can unreliable auditory hazard warnings help the driver? The effect of timing errors and false alarms on road hazard detection in dynamic road scenes.

Supplementary Materials

Full Statistics Tables

Experiment 1

Supplementary Table 1 – All pairwise comparisons of reaction time in each cue timing condition

**Contrast**	**Estimate**	**SE**	**DF**	***t***	***p***
-0.25 v. -0.5	0.104	0.028	235	3.765	0.003
-0.25 v. 0	-0.097	0.028	235	-3.524	0.007
-0.25 v. 0.1	-0.146	0.028	235	-5.314	<.001
-0.25 v no-cue during cue block	-0.270	0.028	235	-9.8	<.001
-0.25 v. no-cue during no-cue block	-0.293	0.028	235	-10.664	<.001
-0.5 v. 0	-0.201	0.028	235	-7.288	<.001
-0.5 v. 0.1	-0.250	0.028	235	-9.079	<.001
-0.5 v. no-cue during cue block	-0.374	0.028	235	-13.565	<.001
-0.5 v. no-cue during no-cue block	-0.397	0.028	235	-14.429	<.001
0 v. 0.1	-0.049	0.028	235	-1.791	0.47
0 v. no-cue during cue block	-0.173	0.028	235	-6.277	<.001
0 v. no-cue during no-cue block	-0.197	0.028	235	-7.141	<.001
0.1 v. no-cue during cue block	-0.124	0.028	235	-4.486	0.002
0.1 v. no-cue during no-cue block	-0.147	0.028	235	-5.35	<.001
no-cue during cued block v. no-cue during no-cue block	-0.024	0.028	235	-0.864	0.95

Supplementary Table 2 – All pairwise comparisons of hazard localization accuracy in each cue timing condition

**Contrast**	**Estimate**	**SE**	**DF**	***t***	***p***
-0.25 v. -0.5	0.009	0.011	235	0.852	0.96
-0.25 v. 0	-0.008	0.011	235	-0.697	0.98
-0.25 v. 0.1	-0.004	0.011	235	-0.441	0.99
-0.25 v no-cue during cue block	-0.02	0.011	235	-1.864	0.43
-0.25 v. no-cue during no-cue block	-0.025	0.011	235	-2.28	0.21
-0.5 v. 0	-0.017	0.011	235	-1.549	0.63
-0.5 v. 0.1	-0.014	0.011	235	-1.293	0.79
-0.5 v. no-cue during cue block	-0.029	0.011	235	-2.716	0.08
-0.5 v. no-cue during no-cue block	-0.034	0.011	235	-3.132	0.02
0 v. 0.1	0.003	0.011	235	0.255	0.99
0 v. no-cue during cue block	-0.013	0.011	235	-1.167	0.85
0 v. no-cue during no-cue block	-0.017	0.011	235	-1.583	0.61
0.1 v. no-cue during cue block	-0.015	0.011	235	-1.422	0.71
0.1 v. no-cue during no-cue block	-0.02	0.011	235	-1.838	0.44
no-cue during cued block v. no-cue during no-cue block	-0.005	0.011	235	-0.416	0.99

Experiment 2

Supplementary Table 3. Results of ANOVAs on reaction time and accuracy for hazard present and absent trials

	Effect	DF1	DF2	*F*	*p*	η^2_G	*W*	*p*	ε_{GG}	p_{GG}
**Hazard Present Trials**										
RT	Reliability	1	46	0.163	0.69	0.003				
	Cue Validity	2	92	2.477	0.09	0.008	0.973	0.54	0.974	0.09
	Reliability × Cue Validity	2	92	0.593	0.55	0.002	0.973	0.54	0.974	0.55
Accuracy	Reliability	1	46	0.701	0.41	0.01				
	Cue Validity	2	92	1.277	0.28	0.009	0.764	0.002	0.81	0.28
	Reliability × Cue Validity	2	92	0.404	0.67	0.003	0.764	0.002	0.81	0.63
**Hazard Absent Trials**										
RT	Reliability	1	46	0.384	0.54	0.006				
	Cue Validity*	2	92	46.331	< 0. 001	0.226	0.941	0.25	0.944	< 0.001
	Reliability × Cue Validity	2	92	1.055	0.35	0.007	0.941	0.25	0.944	0.35
	**Simple Main Effects Analysis**									
	Valid v. Invalid*	1	47	82.339	< 0.001	0.247				
	Invalid v. No-cue baseline*	1	47	50.236	< 0.001	0.232				
	Valid v. No-cue baseline	1	47	0.779	0.38	0.003				
Accuracy	Reliability	1	46	0.08	0.78	0.001				
	Cue Validity*	2	92	3.761	0.03	0.043	0.95	0.31	0.952	0.03
	Reliability × Cue Validity	2	92	2.15	0.12	0.025	0.95	0.31	0.952	0.12
	**Simple Main Effects Analysis**									
	Valid v. Invalid	1	47	3.085	0.09	0.024				
	Invalid v. No-cue baseline	1	47	0.912	0.34	0.008				
	Valid v. No-cue baseline*	1	47	5.964	0.02	0.055				

*Indicates significant tests at $p < 0.05$

Supplementary Table 4. Results of ANOVA of effects of hazard ratings and cue validity on hazard detection performance

Effect	DF1	DF2	*F*	*p*	η^2_G	*W*	*p*	ε_{GG}	p_{GG}
**RT**									
Reliability	1	46	0.275	0.6	0.005				
Cue Validity	2	92	0.954	0.39	0.002	0.985	0.72	0.985	0.39
Rating*	1	46	28.564	< 0.001	0.029				
Reliability × Cue Validity	2	92	0.777	0.46	0.002	0.985	0.72	0.985	0.46
Reliability × Rating	1	46	0.197	0.66	< 0.001				
Cue Validity × Rating	2	92	2.524	0.09	0.004	0.891	0.07	0.901	0.09
Reliability × Cue Validity × Rating	2	92	0.582	0.56	0.001	0.891	0.07	0.901	0.54
**Accuracy**									
Reliability	1	46	0.83	0.37	0.009				
Cue Validity	2	92	1.246	0.29	0.006	0.801	0.007	0.834	0.29
Rating*	1	46	140.891	< 0.001	0.242				
Reliability × Cue Validity	2	92	0.644	0.53	0.003	0.801	0.007	0.834	0.5
Reliability × Rating	1	46	0.095	0.76	< 0.001				
Cue Validity × Rating	2	92	0.318	0.73	0.001	0.631	< 0.001	0.731	0.66
Reliability × Cue Validity × Rating	2	92	0.991	0.38	0.005	0.631	< 0.001	0.731	0.35

*Indicates significant tests at $p < 0.01$

Bayesian model fitting to evaluate the null effect of cue validity

To further evaluate the reliability of the null cue validity result in Experiment 2, we used a Bayesian statistics the confirm the reliability of the null effect. We extracted the trials on which hazards were present and we fitted a Bayesian regression model to localization accuracy and detection response time separately, using the rstanarm package in R. Each model was fitted using 10 chains and 5000 iterations, with 1000 warmup iterations. Default priors were used. Then we compared the posterior distribution of this model against a point null of 0 using the Savage-Dickey density ratio. This procedure resulted in a Bayes Factor (BF_{10}) of 0.053 for localization accuracy, and 0.081 for response time, indicating that the evidence for the null hypothesis is stronger than the evidence for the alternative hypothesis by 19, and 12 times, respectively. For both measures, the evidence supports the null hypothesis.

Analyses on Order Effects

We were interested in a block order effect because cue present and absent conditions were separated into two blocks. The order of the blocks may interact with the strength of the effect of cues. Participants may be more tired during the second block, and so alerting effects of cues should be larger when presented in the second block compared to the first block.

To investigate possible order effects, we conducted 2×3 two-way mixed ANOVAs on RT and accuracy (cue order: cue present block first or cue present block second; cue validity: valid, invalid, or no-cue). Because the effects of valid and invalid cues may be different, we analyzed them separately to examine whether block order modulated the cue validity effect. Although we pre-registered a cue present vs. absent analysis, because we hypothesized that valid and invalid cues may have the opposite effect compared to baseline, an interaction between cue validity and cue block may be obscured if valid and invalid cues were combined together. Therefore, valid and invalid cues were investigated separately.

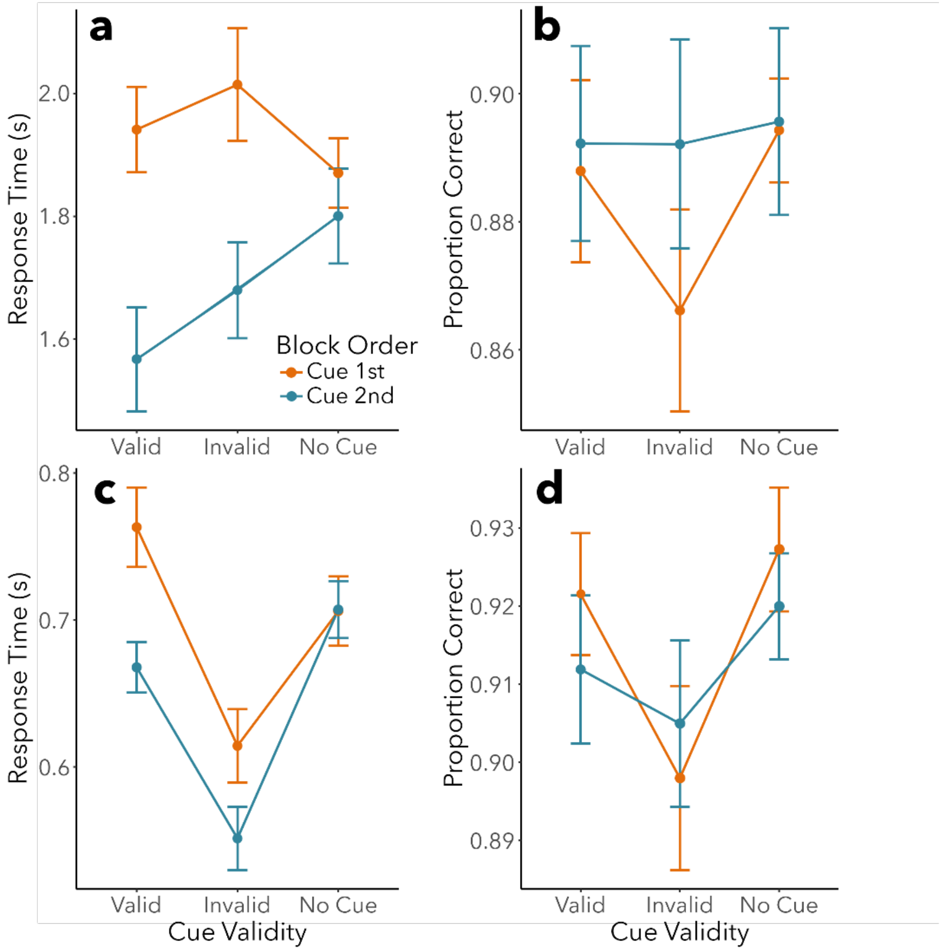

Supplementary Figure 1. Hazard localization response time (a) and accuracy (b) as a function of cue validity and block order for hazard-present trials. Orange represents participants for whom cues occurred in the first block, and blue represents participants for whom cues occurred in the

second block. The bottom two figures show hazard detection response time (c) and accuracy (d) as a function of cue validity and block order for hazard absent trials. Figure conventions are same as in Supplementary Figure 1a and A1b.

Supplementary Figure 1a shows hazard localization response time as a function of cue validity and block order for hazard-present trials. Visually inspecting Supplementary Figure 1a suggests that although the effect of cue validity is similar for both block orders, overall response time is quicker in the cue condition than the no-cue baseline when cues were presented in the second block compared to when cues were presented in the first block. The 2 (Block order) x 3 (Cue validity) ANOVA on RT found significant effects of block order ($F(1,46) = 6.66$, $p = 0.013$, $\eta^2_G = 0.11$), cue validity ($F(2,92) = 3.80$, $p = 0.026$, $\eta^2_G = 0.012$), and a significant block order x cue validity interaction ($F(2,92) = 10.17$, $p < 0.001$, $\eta^2_G = 0.032$).

To further investigate the two-way interaction, we conducted simple main effects analyses of cue validity for each block order. The simple main effect of cue validity was significant for both block orders (cue block 1st: $F(2,46) = 3.59$, $p = 0.035$, $\eta^2_G = 0.027$; cue block 2nd: $F(2,46) = 10.93$, $p < 0.001$, $\eta^2_G = 0.058$). However, the effect was almost twice as large when the cue block was completed second. Further pair-wise comparison indicated that this effect was primarily driven by the fact that when cues were presented in the first block, both cued conditions had slower response times compared to the no-cue baseline (valid: mean difference = 0.07, $t(46) = 1.32$, $p = 0.39$, invalid: mean difference = 0.14 ,$t(46) = 2.68$, $p = 0.03$), although only the invalid condition was significant. However, when cues were presented in the second block, both cued conditions were significantly faster than the no-cue baseline (valid: mean difference = -0.223, $t(46) = -.68$, $p < 0.001$; invalid: mean difference = -0.121, $t(46) = -2.42$, $p = 0.05$). Finally, the difference between valid and invalid cue conditions is slightly larger when the cue were presented in the second block (mean difference = -0.11, $t(46) = -2.255$, $p = 0.07$) than when the cue were presented in the first block (mean difference = -0.073, $t(46) = -1.36$, $p = 0.33$) although none of the effects were significant. These results indicate that participants tend to respond faster during the second block, and fatigue was not a contributor to performance in the second block.

Supplementary Table 5. Results of The 2 (Block order) x 3 (Cue validity) ANOVA on accuracy on hazard present trials

**Effect**	**DF1**	**DF2**	**F**	**p**	η^2_G
Block Order	1	46	0.41	0.52	0.006
Cue Validity	2	92	1.29	0.28	0.009
Block Order × Cue Validity	2	92	0.89	0.41	0.006

Supplementary Figure 1b shows hazard localization accuracy on hazard-present trials as a function of cue validity and block order. Visual inspection of Supplementary Figure 1b suggests that hazard localization accuracy may be lower for invalid cues when cues were presented in the first block compared to all other conditions, but no effects reached statistical significance in the 2 x 3 ANOVA on accuracy ($F < 1.29$, $p > 0.28$, $\eta^2_G < 0.01$; see Supplementary Table 5 for full statistics).

Supplementary Figure 1c shows response time on hazard-absent trials as a function of cue validity and block order. Inspection of Supplementary Figure 1c suggests that invalid cues speeded response times for both block orders, however, the speed of all cued trials was faster when cues were presented in the second block compared to in the first block. However, the RT of no-cue trials remained similar regardless of block order. The results of the 2 x 3 ANOVA on RT corroborated these observations. There was a significant main effect of cue validity ($F(2,92) = 52.36$, $p < 0.001$, $\eta^2_G = 0.056$) , but not of block order ($F(1,46) = 3.73$, $p = 0.06$, $\eta^2_G = 0.24$). There also was a significant interaction between block order and cue validity ($F(2,92) = 5.74$, $p = 0.004$, $\eta^2_G = 0.033$). In the cue 2nd condition, RT for both valid and invalid cue conditions were significantly lower than the no cue baseline (valid: mean difference = -0.39, $t(46) = -2.45$, $p = 0.05$; invalid: mean difference = -0.15, $t(46) = -9.72$, $p < 0.001$).However, in the cue 1st condition, invalid trials were significantly lower than the no-cue condition (mean difference = -0.92, $t(46) = -3.80$, $p = 0.001$), but valid trials were higher than the no-cue condition, although the difference did not reach statistical significance (mean difference = 0.06, $t(46) = 2.37$, $p = 0.06$). The two-way interaction was primarily driven by these different patterns of the cue validity effect between cue 1st and cue 2nd condition. Finally, the difference between valid and invalid cue conditions were comparable for the two block orders, although the cue 1st condition (estimated difference = 0.15, $t(46) = 6.17$, $p < 0.001$) was slightly larger than the cue 2nd condition (estimated difference = 0.12, $t(46) = 7.26$, $p <0.001$).

Supplementary Figure 1d shows accuracy on hazard-absent trials as a function of cue validity and block order. Visual inspection suggest that accuracy increased when cues were invalid compared to valid cues and cue absent blocks. The ANOVA corroborated these observations and found a significant main effect of cue validity ($F(2,92) = 3.63$, $p = 0.03$, $\eta^2_G = 0.04$), but the main effect of block order($F(1,46) = 0.14$, $p = 0.71$, $\eta^2_G = 0.001$) and the block order x cue validity interaction ($F(2,92) = 0.57$, $p = 0.57$, $\eta^2_G = 0.006$) were not significant. These results suggest that for hazard absent trials, the order effect mainly affect RT but not accuracy.

Analysis on Video Hazardousness

Given that not all hazards are the same, cues may confer more benefit for hazard localization when the road situation is more ambiguous. The Road Hazard Stimuli used in this study also included hazardousness ratings from a separate sample of 48 drivers who rated the hazardousness of each video on a continuous slider from 0 (not at all hazardous) to 1 (extremely hazardous). Low-rated hazards are less hazardous and may be less noticeable than high-rated hazards, and we expected cues to especially benefit responses to ambiguous hazards. Unlike the other analyses reported in this study, the analysis involving hazardousness was not pre-registered.

To examine the idea that the detection of more ambiguous hazards, or hazards that are relatively less dangerous benefit more from warning cues, we conducted separate 2 (hazardousness rating: high and low) ×3 (trial validity: valid, invalid, no cue) within groups ANOVA on RT and detection accuracy. Each hazard video was assigned to the high or low hazardousness rating category based on a median split. Any significant effects involving hazardousness rating would indicate that the validity effect depended on how ambiguous the hazard was.

Supplementary Figure 1a and 1b show the effect of trial validity and reliability on RT for low-rated hazards and high-rated hazards, respectively. Visual inspection of Supplementary Figure 1a and 1b suggests that RT for high-rated hazards is overall faster than for low-rated hazards.

However, RT in cued conditions stayed around the no-cue baseline in all conditions. The 2(Cue reliability) × 3(Trial validity) × 2(Hazardousness) ANOVA on RT corroborated these results. There was a significant effect of hazardousness ($F(1,46) = 28.564$, $p < 0.01$, $\eta^2_G = 0.029$), but no other effects were significant ($F \leq 2.524$, $p \geq 0.09$, $\eta^2_G \leq 0.005$; see Supplementary Table 4 for full statistics).

Supplementary Figure 1

Hazard Detection Performance as a Function of Trial validity, Reliability, and Hazard Rating

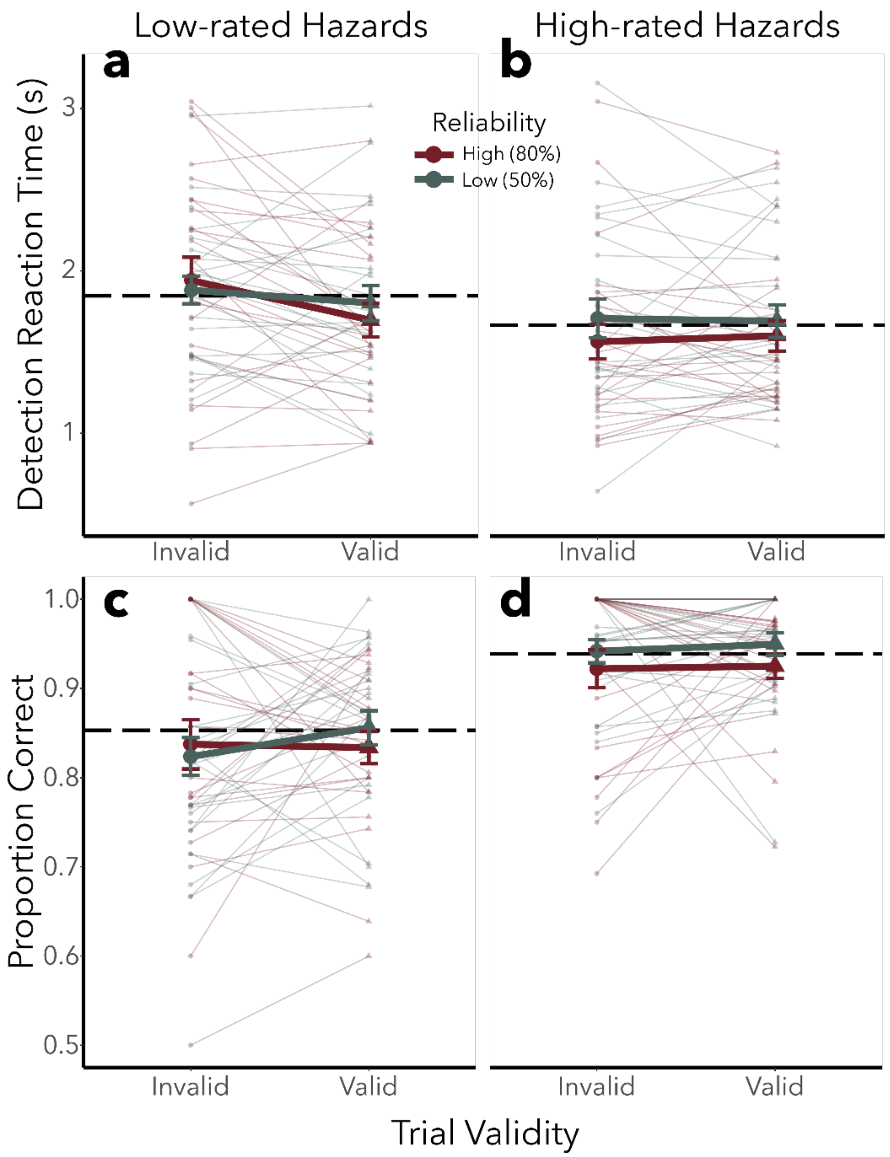

Hazard detection response time for correctly localized hazards as a function of trial validity and reliability for low-rated (a) and high-rated hazards (b). Hazard localization accuracy as a function of trial validity and reliability for low-rated (c) and high-rated (d) hazards. Figure conventions are identical as Figure 4 in the manuscript. Overall reaction time was faster for high-rated hazards

than for low-rated hazards, and proportion correct was also higher for high-rated hazards than low-rated hazards. Although visual inspection of the figures suggests that the interaction between cue reliability and validity may differ between low-rated hazards and high-rated hazards, these effects were not statistically significant.

Supplementary Figure 1c and 1d show the effect of trial validity and reliability on accuracy for low-rated hazards and high-rated hazards, respectively. Inspection of Supplementary Figure 1c and 1d indicates that accuracy results are largely consistent with those of RT. Accuracy stayed near baseline in cued conditions, however, overall accuracy is higher for high-rated hazards than low-rated hazards. Consistent with these observations, the 2(Cue reliability) × 3(Trial validity) × 2(Hazardousness rating) ANOVA on accuracy found a significant main effect of hazardousness rating ($F(1,46) = 140.891$, $p < 0.001$, $\eta^2_G = 0.242$) but no other effects were significant ($F \leq 1.246$, $p \geq 0.29$, $\eta^2_G \leq 0.009$; see Supplementary Table 4 for full statistics). Taken together, these results indicate that high-rated hazards were detected significantly faster and with higher accuracy compared to low-rated hazards. However, hazardousness rating did not significantly modulate the effect of cueing.

The analysis using videos split by hazardousness ratings found little evidence that drivers benefit more from cues when the hazard was ambiguous. One may argue that low-rated hazards are not more ambiguous, but simply less dangerous. However, observers responded to low-rated hazards more slowly and less accurately, suggesting that participants needed more time to locate the hazard and perhaps needed more information than what was available in these videos. These observations are consistent with the idea that low-rated hazards were more difficult to localize than high-rated hazards, rather than simply less dangerous. However, these results suggest that hazardousness did not modulate the effect of cueing.
